# Cross-talk between Bcr-abl and the Thioredoxin System in Chronic Myeloid Leukaemia: Implications for CML Treatment

**DOI:** 10.3390/antiox9030207

**Published:** 2020-03-03

**Authors:** Erin Clapper, Sicong Wang, Prahlad V. Raninga, Giovanna Di Trapani, Kathryn F. Tonissen

**Affiliations:** 1 School of Environment and Science, Griffith University, Nathan, Brisbane, QLD 4111, Australia; e.clapper@griffith.edu.au (E.C.); sicong.wang@griffith.edu.au (S.W.); 2 Griffith Institute for Drug Discovery, Griffith University, Nathan, Brisbane, QLD 4111, Australia; 3 Signal Transduction Laboratory, QIMR Berghofer Medical Research Institute, Herston, Brisbane, QLD 4006, Australia; prahlad.raninga@qimrberghofer.edu.au

**Keywords:** CML, bcr-abl, thioredoxin, ROS, apoptosis, imatinib, drug resistance

## Abstract

Chronic myeloid leukaemia (CML) is currently treated with inhibitors of the CML specific oncoprotein, bcr-abl. While this strategy is initially successful, drug resistance can become a problem. Therefore, new targets need to be identified to ensure the disease can be appropriately managed. The thioredoxin (Trx) system, comprised of Trx, thioredoxin reductase (TrxR), and NADPH, is an antioxidant system previously identified as a target for therapies aimed at overcoming drug resistance in other cancers. We assessed the effectiveness of TrxR inhibitors on drug resistant CML cells and examined links between TrxR and the bcr-abl cell-signalling pathway. Two TrxR inhibitors, auranofin and [Au(d2pype)_2_]Cl, increased intracellular ROS levels and elicited apoptosis in both sensitive and imatinib resistant CML cells. Inhibition of TrxR activity by these pharmacological inhibitors, or by specific siRNA, also resulted in decreased bcr-abl mRNA and protein levels, and lower bcr-abl downstream signalling activity, potentially enhancing the effectiveness of TrxR inhibitors as CML therapies. In addition, imatinib resistant CML cell lines showed upregulated expression of the Trx system. Furthermore, analysis of datasets showed that CML patients who did not respond to imatinib had higher Trx mRNA levels than patients who responded to treatment. Our study demonstrates a link between the Trx system and the bcr-abl protein and highlights the therapeutic potential of targeting the Trx system to improve CML patients’ outcomes.

## 1. Introduction

Chronic myeloid leukaemia (CML) is a myeloproliferative disorder caused by a fusion oncoprotein known as bcr-abl. The bcr-abl fusion arises from a reciprocal chromosomal translocation between chromosomes 9 and 22, which results in a truncated chromosome 22 known as the Philadelphia chromosome. Bcr-abl is a constitutively active non-receptor tyrosine kinase with the ability to over-activate various cell survival pathways such as the AKT and JAK/STAT pathways [1]. There is evidence that the transcription of the bcr-abl gene is controlled via the MYC pathway [2,3]. Studies have also shown that the MYC oncogene is upregulated in CML, which potentially could lead to the increased aggression of the disease, and lack of response to treatment [4,5].

Currently, the vast majority of CML therapies are tyrosine kinase inhibitors (TKIs) that target bcr-abl. While these therapies are effective in the chronic phase of the disease, the final and most severe stage of this disease, blast crisis, is still untreatable with chemotherapy [6,7]. Furthermore, drug resistance is becoming a major issue in the treatment of CML. Since most CML treatments are bcr-abl specific, this results in high levels of acquired drug resistance, due primarily to mutations within bcr-abl [8,9,10]. Some mutations, such as the T315I mutation, significantly reduce the efficacy of TKIs, including imatinib, the primary CML treatment. Chahardouli et al. [11] found that this mutation alone was present in 7% of CML patients examined in their studies, meaning that this entire sub section of patients would be highly resistant to imatinib. In addition, there are many other bcr-abl mutations that result in TKI resistance [12]. To overcome this resistance, bcr-abl independent treatments must be established.

The thioredoxin (Trx) system is currently being studied as a novel target in overcoming drug resistance in other cancers, such as multiple myeloma and breast cancer [13,14,15,16,17,18,19]. The Trx system is an antioxidant system that is involved in maintaining low levels of reactive oxygen species (ROS), as well as activating various cell survival pathways, such as the NF-κB and the hypoxia inducible factor 1 (HIF1) pathways. Upregulation of both these pathways have previously been associated with poor prognosis in other cancers [20,21]. Components of the Trx system (Trx1 and thioredoxin reductase 1 (TrxR1)) are regulated by the Nrf-2 transcription factor and are highly expressed in various cancers including blood cancers, such as multiple myeloma [19] and lymphoma [22]. This gives these cancers a survival advantage, but also presents a novel target in overcoming drug resistance. Conversely, the Trx system is also mediated by thioredoxin interacting protein (TXNIP), which is able to directly bind to the active site of Trx and prevent its function, thereby decreasing the survival advantage provided by the Trx system [23]. Therefore, this gene is considered to be a tumour suppressor and high levels of TXNIP expression are associated with a positive prognosis [24,25]. 

Inhibitors of the Trx system are currently being investigated as novel targets in cancer treatment [18,26,27]. Specifically, these compounds target the activity of TrxR. Many TrxR inhibitors are transition metal based, with the most commonly used metal being gold [28,29,30]. The transition metal ion is able to bind to the selenocysteine residues in the active site of the TrxR protein, which therefore prevents its function [31,32]. A commonly used TrxR inhibitor is the FDA approved anti-rheumatic gold-based compound auranofin. It has been demonstrated that the gold atom in auranofin is able to directly bind to the selenium residue in the active site of TrxR [33,34]. Auranofin has been shown to have anti-cancer properties in breast and ovarian cancers [35,36], and is currently in clinical trials for lung cancer (NCT01737502) and ovarian cancer (NCT03456700). However, more specific TrxR inhibitors are also in development; a notable example is [Au(d2pype)_2_]Cl [30], which will also be used in this study. This TrxR inhibitor has been shown to be effective in inducing cell death in breast cancer [30] and multiple myeloma [13] cells. This study aims to investigate TrxR as a possible therapeutic target by assessing the effectiveness of TrxR inhibitors on CML cells and on overcoming TKI resistance, as well as studying a potential cross talk between bcr-abl and the Trx system. This study identifies a link between these two systems, and this may have strong implications for CML treatment.

## 2. Materials and Methods 

### 2.1. Cell Lines and Reagents

The K562 and KU812 CML cell lines were used in this project. Both cell lines are well studied and documented [37,38], and were taken from patients in blast crisis. The K562 cells (ATCC^®^ Number: CRL-3343) were a gift from Dr. Chris Slape (Translational Research Institute, QLD, Australia), and the KU812 cell line was purchased from Cell Bank Australia (cat. no 90071807). Cell lines were cultured in RPMI-1640 media (Gibco, Gaithersburg, MD, USA) containing 10% Fetal Bovine Serum (FBS) (Bovagen, France), 200mM L-glutamate, and 100 U/mL penicillin and 100 μg/mL streptomycin (Gibco, Gaithersburg, MD, USA). Both cell lines were authenticated by the Griffith University DNA sequencing facility using the STR profiling method (GenePrint 10 system, Promega, Madison, WI, USA). All oligonucleotides used in RT-qPCR were obtained from Integrated DNA Technologies (IDT, Singapore, Singapore). The β-Tubulin polyclonal antibody (cat no. ab6046) was purchased from Abcam (Cambridge, UK). The vinculin antibody (cat no. 13901) was purchased from Cell Signalling Technology (Davers, MA USA). All other antibodies were purchased from Santa Cruz Biotechnology (Santa Cruz, CA, USA) (c-abl, sc-23; TrxR1, sc-58444; MYC, sc-40; AKT1, sc-5298; p-AKT1, sc-293125). Imatinib (cat. no 220127-57-1) and auranofin (cat. no 34031-32-8) were purchased from Cayman Chemicals (Ann Arbor, MI, USA). [Au(d2pype)_2_]Cl was a gift from Professor Sue Berners-Price (Griffith University, Australia), and was synthesised as described previously [39]. Auranofin and Imatinib were prepared in 100% DMSO at a concentration of 10mM, and [Au(d2pype)2]Cl was prepared in 100% ethanol at a concentration of 8.2 mM. [Au(d2pype)2]Cl was diluted to the required working concentrations in RPMI-1640 media, while auranofin and imatinib were diluted in 1 × phosphate buffered saline (PBS). All chemicals were purchased from Sigma-Aldrich (Sydney, NSW, Australia) unless otherwise stated.

### 2.2. Cell Viability Assay

Cell viability was measured using the Trypan Blue exclusion method, as detailed by Raninga et al. [40]. Briefly, 10 µL of cell culture was mixed with 10 µL of 0.4% Trypan Blue solution. Viable cells were then counted using the Neubauer hemocytometer and an inverted light microscope. Viability of siRNA-transfected cells is presented as a percentage of the number of viable untransfected cells.

### 2.3. Development of Imatinib Resistant CML Cell Lines

To generate imatinib resistant CML cell lines, K562 and KU812 cells were exposed to increasing concentrations of imatinib over an extended period of time in T25 cm^2^ flasks. K562 cells were treated with a starting concentration of 100 nM of imatinib for one week. Following this, cells were resuspended in fresh media and left to recover for a week. The following week, this process was repeated with 200 nM of imatinib and then cells were progressively treated for 1 week with 400, 600, 800, and 1000 nM imatinib, with a one-week recovery period in-between each imatinib treatment. The same process was used with the KU812 cells, except the concentrations used were: 20, 50, 100, 200, 300, 400, and 500 nM of imatinib. The degree of imatinib resistance exhibited by the K562R and KU812R cells was tested using MTT proliferation assays (as described in Section 2.5). 

### 2.4. Transient Transfections

K562 cells (2 × 10^6^) were collected and resuspended in pre-transfection media (RPMI-1640 media without FBS or antibiotics) and 100 µl was transferred into transfection cuvettes. Then 50 nM of three distinct TrxR1 siRNA molecules or control siRNA (IDT, Singapore, Singapore) were added to separate cuvettes and incubated for 5 min at room temperature. Cells were transfected using the Amaxa Nucleofector (T-016 program) (Lonza, Basel, Switzerland) and then left to incubate for a further 5 min at room temperature. Cells were then transferred to a 24 well plate in post-transfection media (RPMI-1640 media with 10% FBS and no antibiotics) and incubated for at least 24 h prior to further testing.

### 2.5. MTT Cell Proliferation Assay

MTT proliferation assays were performed using the sensitive and resistant K562 and KU812 cell lines. Initially, 30,000 cells were transferred into a clear-walled 96 well plate in triplicate. Cells were then treated with the compounds at the concentrations specified in each results section. Media was used as a blank. Cells were treated for either 24 or 48 h. After this time 10 μL of 3-(4 5-dimethylthiazol-2-yl)-2 5-diphenyltetrazolium bromide (MTT) reagent was added per well and the plate was incubated for 2–3 h at 37 °C. Following this incubation, 25 μL of 20% SDS/0.01 M HCl was added to each well and left overnight. The plate was read the next day using the FLUOstar Omega plate reader (BMG LabTech, Ortenberg, Germany) at an absorbance of 570 nm.

### 2.6. Caspase-3 Activity Assay

Caspase-3 activity was measured using the protocol described by Cox et al. [41]. K562 cells were transferred to a 24-well plate (1 × 10^6^ cells/well) and treated with auranofin, [Au(d2pype)_2_]Cl or imatinib. The plate was then incubated for 24 h at 37 °C and then cells were washed with 1X PBS once and resuspended in 15 μL of 1 × PBS. This cell suspension was then transferred to a black-walled, clear-bottomed 96-well plate and 85 μL of caspase-3 buffer (5 mM DTT; 100 mM HEPES, pH 7.5; 10% (w/v) Sucrose; 0.1% Nonidet P-40; 50 μM Ac-DEVD-AMC (Cayman Chemicals, Ann Arbor, MI, USA)) was added. The plate was then immediately shaken for 1 min in the FLUOstar Omega plate reader (BMG Labtech, Ortenberg, Germany) and then incubated in this plate reader for 15 min at 37 °C. Fluorescence was measured at Ex_370_/Em_445_.

### 2.7. Thioredoxin Reductase Activity Assay

TrxR activity assays were performed as described previously [13,19,42]. Briefly, 1.5 × 10^6^ cells were treated with auranofin or [Au(d2pype)_2_]Cl for 24 h. Following this, cells were lysed in cell lysis buffer (150 mM NaCl, 50 mM Tris-Cl, pH 8; 0.5% Nonidet P-40, 0.5 mM EDTA, 2 mM PMSF, 1 μL/mL protease inhibitor cocktail VI and 1× PBS) as described by Sze et al. [13]. TrxR activity was then measured by adding 15 µL of whole cell lysate to 25 µL of sample buffer (0.1 M KPi; 20 mM EDTA; 0.1 mg/mL BSA) and 200 µL of assay buffer (0.5 M KPi, pH 7.5; 200 mM EDTA; 20 mM NADPH; 125 mM DTNB). Absorbance was then read at 412 nm every 30 s for 10 min using the SpectraMax M3 plate reader (Molecular Devices, San Jose, CA, USA). Units of TrxR activity was calculated using the extinction co-efficient of TNB (the reduced form of DTNB) at 412 nm (13.6 × 10^3^ M^−1^cm^−1^). The specific TrxR activity (U/mg protein) was calculated by normalizing the units of TrxR activity with the quantity of protein in each sample (as determined using the BioRad DC protein assay reagent (BioRad, Hercules, CA, USA) as per the manufacturer’s instructions).

### 2.8. ROS Levels 

Intracellular ROS levels were measured using a H_2_DCFDA based assay, as detailed by Rushworth et al. [43]. CML cells were transferred to a 24 well plate (1 × 10^6^ cells/well) and treated with TrxR inhibitors for 24 h. After this time, 5 µM of H_2_DCFDA was added to each well and the plate was incubated in the dark at 37 °C for a further 30 min. Cells were then washed with 1 × PBS and resuspended in 200 µL of 1 × PBS. Then 100µl of the suspension was added to a black-walled 96-well plate in duplicate. The fluorescence was read at Em_495_/Ex_515_ in the FLUOstar Omega plate reader (BMG Labtech, Ortenberg, Germany)_._ The total cellular ROS level was calculated relative to the cell number in each well.

### 2.9. Reverse Transcriptase Quantitative PCR (RT-qPCR)

CML cells were seeded into a 24 well plate (0.5 × 10^6^ cells/well) and treated for 24 h. Following this time, RNA was extracted using TriSure (Bioline, Sydney, NSW, Australia) according to the manufacturer’s instructions. Then cDNA was prepared (500 ng/sample) using the GoScript cDNA synthesis kit following the manufacturer’s instructions (Promega, Madison, WI, USA). RT-qPCR was performed using approximately 150 ng of cDNA, 200 nM of forward and reverse primers and 4.5 µL of the SensiFAST SYBR mix (Bioline, Sydney, NSW, Australia) and made up to 10 µL with PCR grade water. RT-qPCR reactions were run on the CFX96 Touch Real-Time PCR Detection System (BioRad, Hercules, CA, USA) using the following cycling parameters: 95 °C for 2 min and 40 cycles of 95 °C for 10 s, 60 °C for 15 s and 72 °C for 20 s. The primers used were: RPL32 forward: 5′ CAG GGT TCG TAG AAG ATT CAA GGG, RPL32 reverse: 5′ CTT GGA GGA AAA CAT TGT GAG CGA TC, Nrf2 forward: 5′ ATT CAG CCA GCC CAG CAC, Nrf2 reverse: 5′ CGA AGA AAC CTC ATT GTC ATC, Trx1 forward: 5′ GCC AGT TTA TAA AGG GAG AGA GCA, Trx1 reverse: 5′ TGA TCA TTT TGC AAG GCC CA, TrxR1 forward: 5′ GGA ATC CAC CCT GTC TCT GC, TrxR1 reverse: 5′ ACG AGC CAG TGG TTT GCA GT, TXNIP forward: 5′ GGC ACC TGT GTC TGC TAA AA, TXNIP reverse: 5′ CGG GAA CAT GTA TTC TCA AA, bcr-abl forward: 5′ CAT TCC GCT GAC CAT CAA TAA G, bcr-abl reverse: 5′ GAT GCT ACT GGC CGC TGA AG. Results were analysed using the ΔΔCq method, and samples were normalised against the housekeeping gene RPL32.

### 2.10. Western Blotting 

Western blotting was performed as described by Karlenius et al. [44]. Cells were seeded into a 6-well plate (1.5 × 10^6^ cells/well) and incubated with or without TrxR inhibitors for 24 h. Whole cell lysates were prepared using the NP-40 lysis buffer as described in Section 2.7. Then, 80 µg of cell lysate was loaded and run on SDS polyacrylamide gels of an appropriate percentage for each protein: 8% (bcr-abl), 15% (Trx1) or 10% (all remaining proteins tested). Proteins on the gel were then transferred to a PVDF membrane using the Bio-Rad TransBlot Turbo apparatus, as per the manufacturer’s instructions. Blots were blocked in 5% blotto and incubated overnight with the relevant primary antibody at 4 °C. Blots were then incubated in the corresponding horse radish peroxidase (HRP) conjugated secondary antibody (BioRad, Hercules, CA, USA) for two hours at room temperature. Blots were imaged using the GE healthcare enhanced chemiluminescence (ECL) detection kit with the FujiFilm LAS300.

### 2.11. Bioinformatics

Array data was acquired from the GEO database. The GSE2535 dataset [45] was used for this study, and in this dataset mRNA expression had been measured using the Affymetrix Human Genome U95 Version 2 Array. Samples were separated into two groups: CML patients that had normal imatinib response, and CML patients that were resistant to imatinib. The mRNA expression levels for Trx1 and TXNIP were determined and the fold change between the two groups was calculated and graphed using Graphpad Prism 8. In this dataset there were 16 imatinib responder patients and 12 non-responders.

### 2.12. Statistical Analysis

All data in this paper were analysed using the Graphpad Prism 8 software. A *p* value < 0.05 using the appropriate statistical test was considered significant. All graphs are displayed as mean ± SEM.

## 3. Results

### 3.1. Auranofin and [Au(d2pype)_2_]Cl Induce Apoptosis in CML Cells

**Figure 1:**
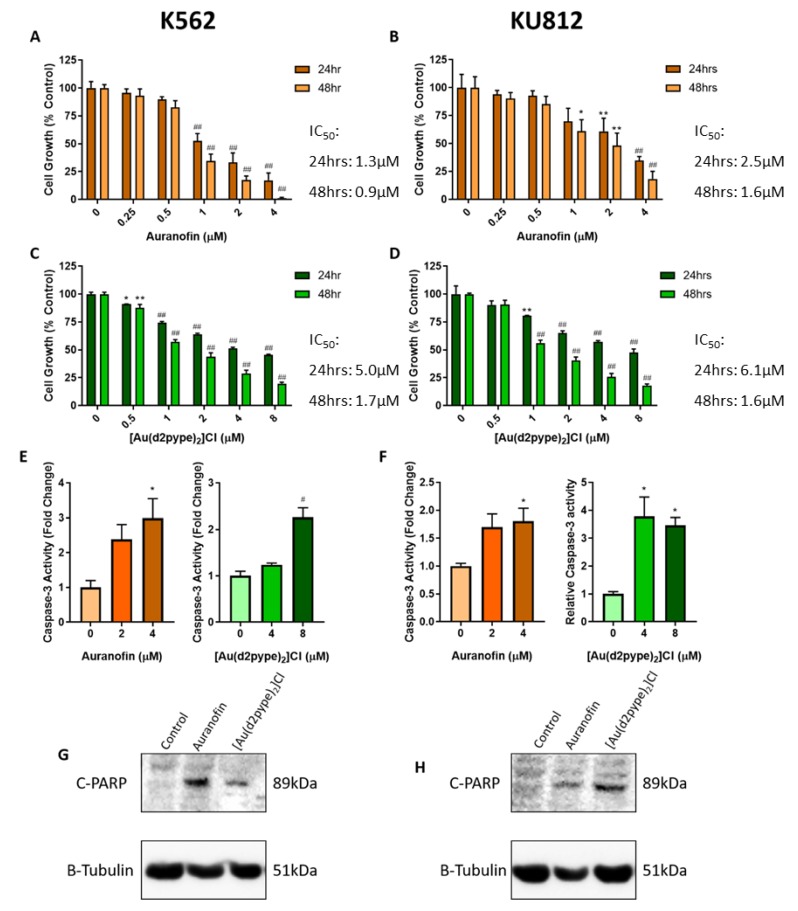
TrxR Inhibitors Reduce Cell Growth and Elicit Apoptosis in CML Cells. A-D: K562 and KU812 cells were treated with auranofin (**A**,**B**) and [Au(d2pype)_2_]Cl (**C**,**D**) respectively for 24 and 48 h. Cell growth was then measured using the MTT proliferation assay. **E**,**F**: K562 and KU812 respectively were treated with auranofin or [Au(d2pype)_2_]Cl for 24 h then caspase-3 activity was measured, using an Ac-DEVD-AMC based fluorogenic assay. **G**,**H**: Both cell lines were treated with 4 µM of either Auranofin or [Au(d2pype)_2_]Cl for 24 h. Western blotting was performed using an antibody specific to cleaved 89kDa PARP-1 (C-PARP). Β-Tubulin was used as a loading control. MTT results were analysed via two-way ANOVA with Dunnett’s post hoc test. Caspase-3 activity was analysed with multiple T-tests. Statistical tests compared data from the treated and untreated cells. * = *p* < 0.05, **= *p* < 0.01, # = *p* < 0.001. ## = *p* < 0.0001. *N* = 3. Values displayed as mean ± SEM.

To measure the effect of the TrxR inhibitors auranofin and [Au(d2pype)_2_]Cl on cell growth, MTT proliferation assays were performed after 24 h and 48 h of treatment. MTT results shown in Figure 1A–D demonstrate that both TrxR inhibitors were able to elicit a significant degree of cell death in both cell lines. Auranofin shows similar effectiveness after both 24 h and 48 h treatment. However, there is a notable increase in the effectiveness of [Au(d2pype)_2_]Cl after 48 h compared to 24 h of treatment. Both TrxR inhibitors have an IC_50_ in K562 and KU812 CML cell lines in the low micromolar range after 48 h. In addition, treatment with 4 µM auranofin for 24 h induced a three-fold increase in caspase-3 activity in K562 cells, and a two-fold increase in KU812 cells (Figure 1E,F). In K562 cells a concentration of 8 µM [Au(d2pype)_2_]Cl was required to significantly increase caspase-3 activity. resulting in an approximate 2.5-fold increase. However, in KU812 cells 4 µM of [Au(d2pype)_2_]Cl resulted in a four-fold increase in caspase-3 activity. These assays showed that both auranofin and [Au(d2pype)_2_]Cl were able to significantly increase caspase-3 activity compared to the untreated control. Moreover, both compounds induced the cleavage of poly [ADP-ribose] polymerase 1 (PARP-1), a classical marker of apoptosis (Figure 1G,H). These results suggest that both auranofin and [Au(d2pype)_2_]Cl cause cell death via apoptosis in both CML cell lines. 

### 3.2. Lowered TrxR Activity Via Auranofin and [Au(d2pype)_2_]Cl Results in Increased ROS

**Figure 2:**
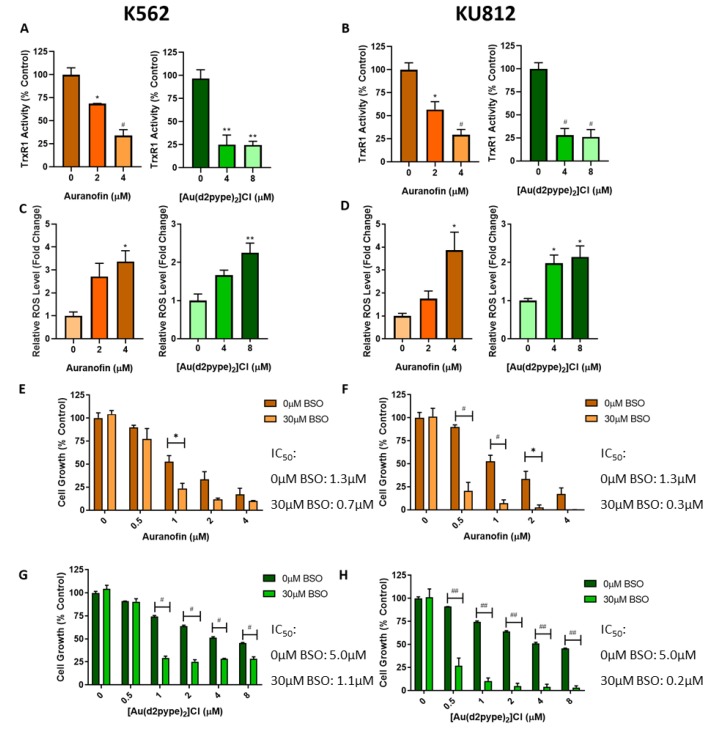
TrxR Inhibitors Decrease TrxR Activity and Induce Higher ROS Levels. **A**,**B**: K562 and KU812 CML cells respectively were treated with either auranofin or [Au(d2pype)_2_]Cl for 24 h. TrxR activity was then measured using a DTNB based colourimetric assay and activity was made relative to total protein. **C**,**D**: K562 and KU812 CML cells respectively were treated with either auranofin or [Au(d2pype)_2_]Cl for 24 h. ROS levels were then measured using a H_2_DCFDA fluorogenic assay. Results were made relative to total cell number. **E**–**H**: Both cell lines were treated with auranofin or [Au(d2pype)_2_]Cl and with or without BSO for 24 h, following this an MTT proliferation assay was performed. Results were analysed with multiple *T*-tests. In figures **A**–**D** statistical tests compared results of untreated and treated cells. In figures E–H statistical tests compared cells treated with or without BSO. * = *p* < 0.05, **= *p* < 0.01, # = *p* < 0.001, ## = *p* < 0.0001. *N* = 3. Values displayed as mean ± SEM.

TrxR activity assays were used to confirm both auranofin and [Au(d2pype)_2_]Cl were able to significantly inhibit TrxR activity after 24 h treatment in K562 (Figure 2A) and KU812 cells (Figure 2B). To assess how this inhibition of TrxR activity affected intracellular ROS levels, the oxidative stress sensitive compound H_2_DCFDA was used. CML cells were treated with auranofin or [Au(d2pype)_2_]Cl for 24 h and ROS levels were measured. Both compounds induced a significantly higher level of ROS in both cell lines compared to untreated cells, although in the KU812 cell line auranofin was more effective at increasing ROS compared to [Au(d2pype)_2_]Cl (Figure 2C,D). 

To assess the impact of the cellular redox state on cell growth, cells were also treated with buthionine sulphoximine (BSO), which inhibits the synthesis of glutathione, the other major cellular antioxidant. A concentration of 30µM BSO does not elicit any quantifiable cell death in either cell line. Cells were then co-treated with 30 µM BSO either with auranofin or [Au(d2pype)_2_]Cl, and in both cases cells underwent significantly more cell death than those treated solely with TrxR inhibitors (Figure 2E–H). The KU812 cells were more sensitive to the BSO/TrxR inhibitor combination compared to the K562 cells. Furthermore, the BSO and [Au(d2pype)_2_]Cl combination was slightly more effective than BSO and auranofin. 

### 3.3. Bcr-abl Expression is Linked to Activity of the Trx System

**Figure 3:**
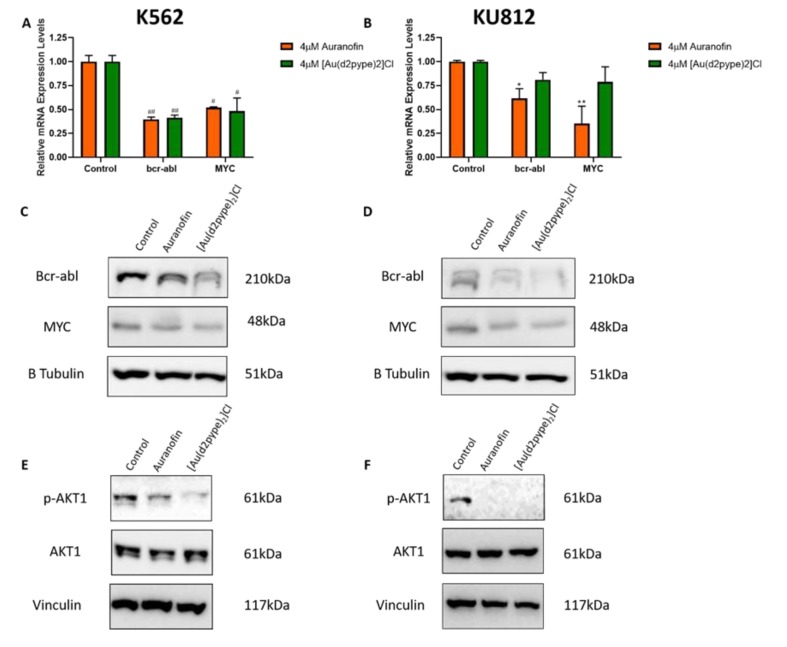
TrxR Inhibitors Reduce mRNA and Protein Expression of the Bcr-abl pathway. **A**,**B**: K562 and KU812 CML cells respectively were treated with 4 µM of either auranofin or [Au(d2pype)_2_]Cl for 24 h. RT-qPCR was then used to measure the mRNA expression levels of bcr-abl and MYC. RPL32 was used as a normaliser **C**,**D**: K562 and KU812 CML cells respectively were treated with 4 µM of either auranofin or [Au(d2pype)_2_]Cl for 24 h. Western blotting was then used to measure bcr-abl and MYC protein levels. **E**,**F**: K562 and KU812 CML cells respectively were treated with either 4µM of auranofin or 8 µM [Au(d2pype)_2_]Cl in the K562 cells and 4 µM of the inhibitors in the KU812 cells for 24 h. Western blotting was then used to measure p-AKT1 and AKT1 levels. Vinculin and Β-tubulin was used as loading controls for western blots. RT-qPCR results were analysed with multiple T-tests. Statistical tests compared results of the treated and untreated cells. * = *p* < 0.05, **= *p* < 0.01, # = *p* < 0.001, ## = *p* < 0.0001. *N* = 3. Values displayed as mean ± SEM.

To investigate the effect of TrxR inhibition on the bcr-abl pathway, the mRNA levels of bcr-abl and MYC, which is involved in the transcription of the bcr-abl gene, were measured using RT-qPCR. In K562 cells (Figure 3A) treatment with both auranofin and [Au(d2pype)_2_]Cl significantly reduced the mRNA expression of both bcr-abl and MYC. However, in KU812 cells (Figure 3B), while auranofin was able to significantly decrease bcr-abl and MYC mRNA expression, [Au(d2pype)_2_]Cl had only a minimal effect on the mRNA expression of both genes. Western blotting of bcr-abl and MYC following auranofin and [Au(d2pype)_2_]Cl treatment was also performed. As seen in Figure 3C,D, auranofin and [Au(d2pype)_2_]Cl treatment markedly decreased both bcr-abl and MYC protein levels after 24 h in both cell lines. In both cell lines it appears that [Au(d2pype)_2_]Cl is more effective in decreasing bcr-abl and MYC protein levels compared to auranofin. Overall, these results imply that the downregulation of the TrxR system via auranofin and [Au(d2pype)_2_]Cl results in decreased expression of bcr-abl and MYC. To confirm bcr-abl pathway inhibition, the phosphorylation level of AKT1, a prominent downstream target of bcr-abl, was assessed using western blotting. As seen in Figure 3E,F, the levels of phosphorylated AKT1 (p-AKT1) are decreased in CML cells treated with both compounds, compared to the untreated control. These results indicate that treatment with either auranofin or [Au(d2pype)_2_]Cl inhibits bcr-abl regulated pathways in CML cells.

### 3.4. Knockdown of TrxR1 in CML cells Results in the Decreased Expression of Bcr-abl

**Figure 4:**
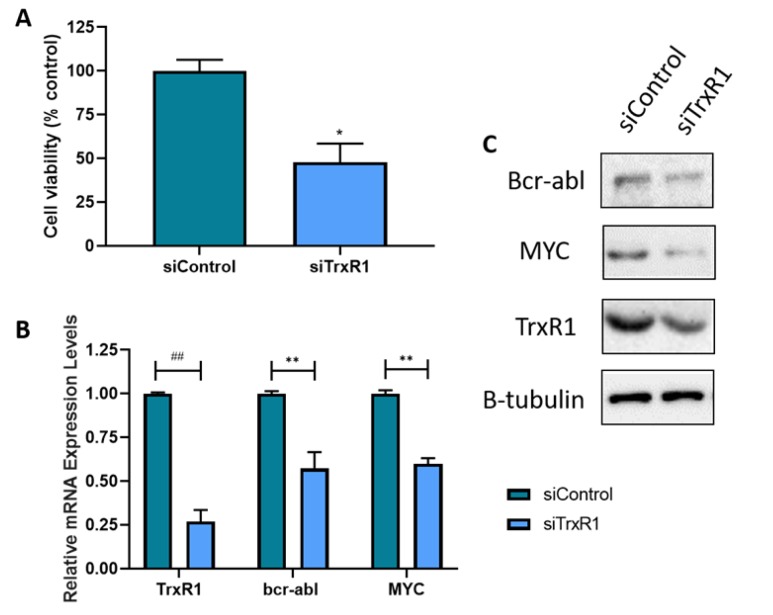
TrxR1 Knockdown Reduces Bcr-abl and c-myc mRNA and Protein Expression. K562 cells were transfected with TrxR1 specific siRNA and a scrambled siRNA control. **A**: Cell viability was then measured after 24 h. **B**: RT-qPCR was performed 24 h after transfection to measure the mRNA expression levels of TrxR1, bcr-abl and MYC. RPL32 was used as a normaliser **C**: Western blotting was performed 48 h after transfection and was used to measure the protein levels of TrxR1, bcr-abl and MYC. Β-tubulin was used as a loading control for western blots. A representative of 3 blots is shown. RT-qPCR and cell viability results were analysed with multiple *T*-tests. Statistical tests compared results of the TrxR1 knocked down cells and the scrambled siRNA control. * = *p* < 0.05, ** = *p* < 0.01, ## = *p* < 0.0001. *N* = 3. Values displayed as mean ± SEM.

To confirm the results observed using chemical inhibitors of TrxR, TrxR1 was knocked down using TrxR1 specific siRNA molecules. Cell viability was measured 24 h after transfection (Figure 4A). Knockdown of TrxR1 resulted in a significant decrease in cell viability when compared to the scrambled siRNA control. This implies that TrxR1 is required for the survival of the CML cells. RT-qPCR was then performed to confirm successful knockdown of TrxR1, as well as to assess bcr-abl and MYC mRNA levels to examine the effect of the TrxR1 knockdown on this pathway. As shown in Figure 4B the mRNA expression levels of all three genes were significantly decreased in the TrxR1 knocked down samples compared to the control. This result shows that it is likely that the decrease in TrxR1 expression leads to a decrease in the bcr-abl/MYC pathways, which corroborates the results observed with auranofin and [Au(d2pype)_2_]Cl. Western blotting experiments confirmed that TrxR1, bcr-abl and MYC protein levels were also considerably reduced (Figure 4C), providing further evidence that TrxR1 knockdown downregulates the bcr-abl pathway.

### 3.5. Imatinib Resistant CML Cells have Upregulated Levels of the Trx System

**Figure 5:**
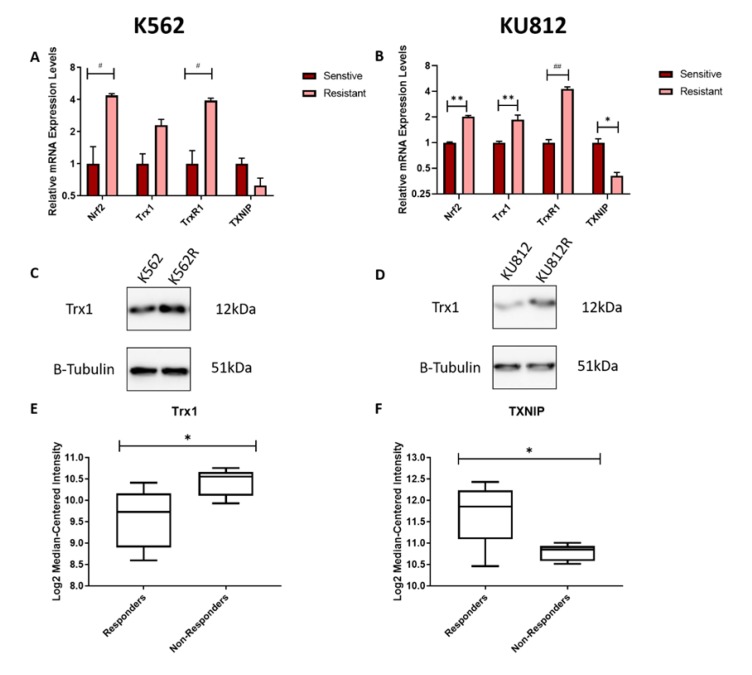
Imatinib Resistant CML cells have Upregulated mRNA and Protein Expression of the Trx System. **A**,**B**: mRNA expression levels of Nrf2, Trx1, TrxR1, and TXNIP were measured using RT-qPCR in sensitive and Resistant K562 and KU812 CML cells respectively, with the sensitive cells being used as a control. RPL32 was used as a normaliser. **C**,**D**: Protein levels of Trx1 were measured in sensitive and resistant K562 and KU812 CML cells respectively. B-tubulin was used as a loading control. **E**,**F**: Database analysis of the mRNA expression levels of Trx1 and TXNIP respectively were compared against samples from CML patients who showed a response to imatinib and those that did not (GSE2535). RT-qPCR results were analysed via two-way ANOVAs with Dunnett’s post hoc test. Database analysis results were analysed via an unpaired T-test. Statistical tests compared results between the sensitive and resistant cell lines. * = *p* < 0.05, ** = *p* < 0.01, # = *p* < 0.001, ## = *p* < 0.0001. *N* = 3 for RT-qPCR and western blotting, *N* = 16 for bioinformatics. Values displayed as mean ± SEM.

Imatinib resistant variants (K562R and KU812R) of the K562 and KU812 cell lines respectively, were developed by treating cells with increasing concentrations of imatinib over an extended period of time. The mRNA expression levels of the Trx system members (trx1 and TrxR1) and regulators (Nrf2 and TXNIP) in imatinib resistant and sensitive CML cells were measured using RT-qPCR. A significantly increased level of Nrf2 and TrxR1 mRNA expression in the K562R cells compared to the K562 cells was observed (Figure 5A). There is also a visible increase in the mRNA expression of Trx1, as it is over two times more highly expressed in the imatinib resistant cells compared to the sensitive parental cell line. Furthermore, there is a visible, but not significant, decrease in the mRNA expression of TXNIP in the resistant K562 cells, compared to the sensitive cells. The KU812 and KU812R RT-qPCR results are shown in Figure 5B. A significant increase in the mRNA expression level of Nrf2, Trx1 and TrxR1 and a significant decrease in the mRNA expression levels of TXNIP were observed, consistent with an overall upregulation of the Trx system in the imatinib resistant CML cells compared to the parental cell lines. 

The protein levels of Trx1 in the K562 and K562R cell lines were measured using western blotting. In both cell lines there is visibly more Trx1 in the resistant variant compared to the sensitive cells (Figure 5C,D). Therefore, both the mRNA and protein expression results show that there is an increase in the level of the Trx system in the K562R and KU812R cell lines compared to the parental cell lines, which may be one of the causes of their increased resistance to TKIs. Bioinformatics analysis of CML patient data was then performed using the GSE2535 database, which contains RNAseq data of CML patients who responded to imatinib and those who did not. Our analysis compared the expression levels of Trx1 and TXNIP between these two groups (Figure 5E,F). A significant increase in the mRNA expression levels of Trx1 and a significantly decreased level of TXNIP in the imatinib non-responders was observed, which together with the above results further links the Trx system as a contributing factor to the ineffectiveness of imatinib in resistant cells.

### 3.6. Imatinib Resistant CML Cells are Sensitive to TrxR Inhibitors

**Figure 6:**
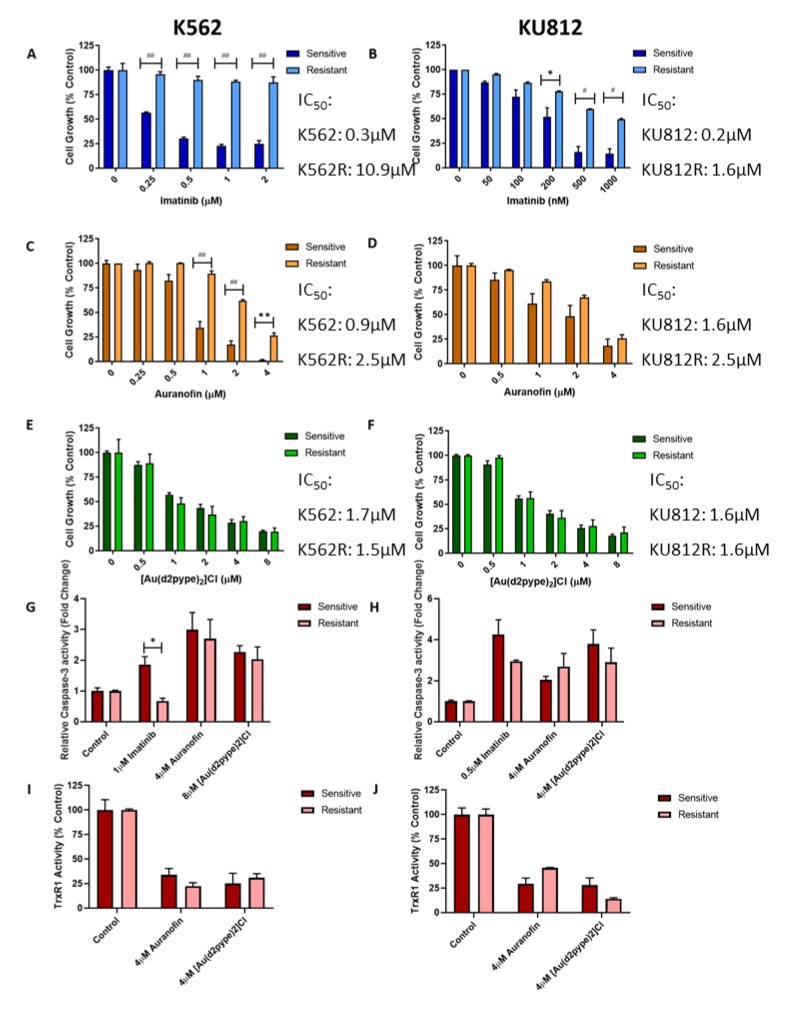
Imatinib Resistant CML cells are susceptible to TrxR Inhibitors. **A**–**F**: Sensitive and resistant K562 and KU812 cells were treated with either imatinib (**A**,**B**), auranofin (**C**,**D**) or [Au(d2pype)_2_]Cl (**E**,**F**) for 48 h. Following all these treatment times MTT proliferation assays were performed. **G**,**H**: Caspase-3 activity assays were performed on sensitive and resistant K562 and KU812 cells after 24 h treatment of either imatinib, auranofin or [Au(d2pype)_2_]Cl. **I**,**J**: TrxR activity assays were performed on sensitive and resistant K562 and KU812 cells after 24 h treatment of either auranofin or [Au(d2pype)_2_]Cl. Results were analysed via two-way ANOVAs with a Dunnett’s post hoc test. Statistical tests compared results between the sensitive and resistant cell lines. * = *p* < 0.05, ** = *p* < 0.01, # = *p* < 0.001, ## = *p* < 0.0001. *N* = 3. Values displayed as mean ± SEM.

MTT cell proliferation assays were used to determine the sensitivity of the K562 and K562R cell lines to imatinib and TrxR inhibitors. The IC_50_ of imatinib in the K562 cells was 0.3 µM, whereas in the resistant K562R cells it was 10.9 µM (Figure 6A), indicating that the resistant cells are 37 times less responsive to imatinib than the parental cell line. For the KU812 cell line, Figure 6B shows the IC_50_ of imatinib in the parental KU812 cell line was approximately 0.2 µM, while in the KU812R cells imatinib had an IC_50_ of approximately 1.6 µM, which is eight times higher. 

Both K562R and KU812R cells were then exposed to auranofin and [Au(d2pype)_2_]Cl, and these results were compared to those of the parental cell lines (Figure 6C–F). The K562R cell line exhibited a degree of resistance towards auranofin compared to the parental cell line (Figure C,D). The IC_50_ of auranofin is approximately 0.9 µM after 48 h in the parental cell line, but is approximately 2.5 µM in the resistant cell line, indicating that this drug is about 3× less effective in the K562R cells. However, these results show that auranofin is still effective in K562 cells that are no longer responsive to imatinib. In the KU812R cell line, there is no significant difference in the efficacy of auranofin compared to the sensitive KU812 cells. 

The [Au(d2pype)_2_]Cl compound showed almost identical effectiveness on imatinib resistant and sensitive CML cells (Figure 6E,F). There was no statistical significance between the cell growth between the sensitive and resistant cell lines at any tested concentration of [Au(d2pype)2]Cl. In fact, the IC_50_ of [Au(d2pype)_2_]Cl in K562R cells after 48 h was approximately 1.5 µM, while in the sensitive K562 cells it was 1.7 µM, making the K562R cells slightly more sensitive to [Au(d2pype)2]Cl compared to the parental cell line. The IC_50_ values of [Au(d2pype)_2_]Cl in both the KU812 and KU812R cell lines after 48 h were approximately 1.6 µM, demonstrating the effectiveness of [Au(d2pype)2]Cl was not affected by imatinib resistance. Therefore, of the two TrxR inhibitors [Au(d2pype)_2_]Cl was more effective in overcoming imatinib resistance in CML cells, although both compounds to some degree were able to inhibit growth of imatinib resistant CML cells. 

Caspase-3 activity was measured in both sensitive and resistant K562 and KU812 cells treated with imatinib, auranofin or [Au(d2pype)_2_]Cl for 24 h (Figure 6G,H). Imatinib induced a significantly lower caspase-3 activity level in resistant K562R cells and a visibly lower level in the resistant KU812R cells, compared to their parental sensitive cell lines. In contrast, there was no significant difference in the caspase-3 activity between the sensitive and resistant cells treated with both TrxR inhibitors, implying that the imatinib resistance phenotype has no significant effect on the ability of TrxR inhibitors to induce apoptosis in CML cells. TrxR activity assays, shown in Figure 6I,J, show that both auranofin and [Au(d2pype)_2_]Cl are still able to decrease TrxR activity to the same level in the resistant cell lines as they do in the parental cell lines, demonstrating their effectiveness as TrxR inhibitors in imatinib resistant CML cells. 

## 4. Discussion

The Trx system is a prominent antioxidant system in the body that is responsible for maintaining healthy levels of ROS and activating various cell survival pathways. Previous studies have demonstrated the effectiveness of gold based TrxR inhibitors on overcoming various forms of drug resistance in cancer [19,46,47]. This study aimed to examine the effectiveness of two TrxR1 inhibitors, auranofin and [Au(d2pype)_2_]Cl, on overcoming intrinsic imatinib resistance in CML and to investigate the possible links between the CML causing protein, bcr-abl, and the Trx system. It has previously been shown that neither of these compounds elicited significant cell death in healthy peripheral non-cancerous blood mononuclear cells (PBMCs) [13], indicating the suitability of TrxR inhibitors for treating patients.

This study has shown that auranofin and [Au(d2pype)_2_]Cl were able to decrease cell proliferation in the K562 and KU812 blast crisis CML cell lines. Both compounds were also observed to elicit significantly increased levels of caspase-3 activity, with visible PARP-1 degradation, which suggests that the cells are dying via apoptosis. Similar results were observed with auranofin [16,48] and [Au(d2pype)_2_]Cl [13,30] in other cancer cells. TrxR activity was measured upon treatment of auranofin and [Au(d2pype)_2_]Cl and it was found that both compounds significantly decreased TrxR activity. This result was corroborated by the increased ROS levels present after cells were treated with auranofin and [Au(d2pype)_2_]Cl. The decreased activity of the Trx system caused by these compounds hinders the ability of the cells to regulate ROS levels and thus, upon inhibition of TrxR, an increase in ROS levels was observed. Chen et al. [48] also found that auranofin induced apoptosis in K562 cells in a ROS dependent manner, although, this was not linked to the Trx system. Therefore, it must be acknowledged that some of the results observed with the gold compounds in our study may be partially due to their non TrxR targeted effects [49,50]. However, this increase in ROS may be a mechanism by which TrxR inhibitors induce apoptosis in CML cells. Elevated ROS levels are able to induce apoptosis via a variety of different pathways, for example, the tumour necrosis factor (TNF) and apoptosis signalling kinase (ASK) 1 pathways [51,52]. Furthermore, high ROS levels can also directly damage DNA, lipids and proteins, which also may result in cell death [53]. Co-treatment with the glutathione inhibitor BSO and either TrxR inhibitor showed a synergistic effect, demonstrating that a functional redox state is required for cell viability. Locy et al. [54] and Lu et al. [55] also observed that both these antioxidant systems are crucial for cancer cell survival.

To examine the effect of TrxR inhibition on bcr-abl and its downstream pathways, TrxR was inhibited using not only the chemical inhibitors auranofin and [Au(d2pype)_2_]Cl, but also using specific siRNA knockdown. Inhibition of the Trx system via auranofin, [Au(d2pype)_2_]Cl and by specific siRNA targeting TrxR was observed to result in lowered bcr-abl protein expression. This implies that the decreased activity of the Trx system results in changes to bcr-abl translation or to post-translational modifications that decrease protein levels. This result was supported by RT-qPCR data that showed that treatment of both cell lines with either TrxR inhibitor resulted in significantly decreased mRNA expression levels of bcr-abl. This suggests that the downregulation of the bcr-abl protein by these compounds is through a decrease in transcription. A proposed mechanism of action for this decrease in transcription is through the MYC pathway. MYC is a very prominent oncogene, and is deregulated in many cancers, leading to worsening prognoses due to its involvement in cell proliferation pathways [56,57,58,59]. Deregulation of MYC has also been shown to decrease the effectiveness of imatinib treatment in CML patients [4,5]. This may be due to MYC being able to control the transcription of bcr as well as bcr-abl [2]. Therefore, a decrease in MYC activity would theoretically lead to a decrease in the transcription of bcr-abl. This exact result was observed when CML cells were treated with both TrxR inhibitors, as well as when TrxR1 was directly knocked down. Therefore, it is possible that the downregulation of bcr-abl via TrxR inhibition is through the MYC pathway. It has been previously shown by Sze et al. [13] that treatment of myeloma cells with [Au(d2pype)_2_]Cl resulted in a decrease in MYC mRNA and protein levels, which further supports the hypothesis that the Trx system plays a role in regulating the MYC pathway.

The decrease of bcr-abl expression may be one of the mechanisms by which auranofin and [Au(d2pype)_2_]Cl induce apoptosis, as bcr-abl is responsible for activating many survival pathways in the cell, including the JAK/STAT and AKT pathways. Therefore, a decrease in the activity of these pathways would make cells far more susceptible to treatment. It was shown that treatment with either compound reduced the phosphorylation and thus, activation of AKT1, thereby significantly decreasing the activity of this pathway. The AKT1 pathway is able to induce increased cell growth, survival and metabolism through a large number of downstream pathways including mechanistic target of rapamycin complex (mTORC), p53, and mitogen-activated protein kinase (MAPK) [60]. Therefore, the decrease in the activity of the AKT pathway may very well be a bcr-abl dependant mechanism of TrxR inhibitor induced cell death. However, since this mechanism of bcr-abl targeting is unique from that of bcr-abl specific TKIs, such as imatinib, TrxR inhibitors are still able to induce apoptosis in TKI resistant CML cells.

Acquired TKI resistance is a major hurdle in the treatment of CML, as it is estimated by Angeles-Velazquez et al. [61] that over 20% of all CML patients will become resistant to this class of drug throughout the duration of their battle with CML. Therefore, imatinib resistant variants of the K562 and KU812 cell lines were developed for this project. The K562R cells were approximately 40 times more resistant to imatinib than their sensitive counterpart, while the KU812R cells were around five times more resistant than the sensitive KU812 cells. However, both cell lines were still susceptible to the TrxR inhibitors auranofin and [Au(d2pype)_2_]Cl. This means that the inhibition of TrxR via these two compounds may be able to overcome acquired imatinib resistance in CML. Furthermore, it was observed that the mRNA levels of Nrf2, Trx1 and TrxR1 were significantly higher in the resistant CML cells compared to the parental cells. This increase in antioxidant capability may contribute to the increased resistance towards imatinib in both the K562R and KU812R cell lines. An increase in the expression levels of the Trx system has been linked to drug resistance in several other cancers and is often associated with a poor prognosis [19,62,63]. This increased drug resistance is due to the upregulation of cell survival pathways such as the NF-κB pathway and HIF-1α pathways, which are both regulated by the Trx system [64,65,66,67]. Furthermore, upregulation of Trx also prevents cells from undergoing oxidative stress. TXNIP mRNA expression was significantly decreased, which would likely result in an increase in the activity of the Trx system, as this protein can bind directly to the active site of Trx, and prevent its activity. TXNIP has also been identified as a tumour suppressor, and is often observed to be downregulated in cancer [24,68]. Therefore, the increase in the expression of the Trx system, along with the decreased TXNIP expression observed in the imatinib resistant CML cells are indicative of a more drug resistant cell line. However, auranofin and [Au(d2pype)_2_]Cl are able to overcome this resistance due to their TrxR inhibitory activity.

## 5. Conclusions

In conclusion, the results reported in this paper demonstrate that TrxR is a valid therapeutic target for CML. TrxR inhibitors auranofin and [Au(d2pype)_2_]Cl were able to increase ROS levels and induce apoptosis in CML cell lines. This is important, as novel targets are required in CML treatment since prolonged exposure to TKIs can result in complete resistance to bcr-abl inhibitors. Furthermore, it appears that the Trx system and the transcription of bcr-abl may be linked since targeting TrxR by either chemical inhibitors or specific siRNAs lowered both the mRNA and protein levels of bcr-abl, resulting in decreased activation of AKT1, one of its downstream targets. This would potentially make these treatments even more effective in CML, while still able to overcome TKI resistance. Therefore, it could be suggested that TrxR inhibitors may make suitable treatments or be used in combination therapies for CML patients suffering TKI resistance.
